# Bone formation niche dysfunction in osteoporosis: insights from single-cell and spatial transcriptomic studies

**DOI:** 10.3389/fendo.2026.1909423

**Published:** 2026-08-26

**Authors:** Tingting Tan, Xiaoning Guo, Zhengxiao Ouyang

**Affiliations:** 1 Department of Orthopedics, The Second Xiangya Hospital, Central South University, Changsha, Hunan, China; 2 Department of Immunology, School of Basic Medical Science, Central South University, Changsha, Hunan, China; 3 Osteopathy Laboratory of Surgical Department, The Second Xiangya Hospital, Central South University, Changsha, Hunan, China

**Keywords:** bone formation, bone marrow microenvironment, mesenchymal stromal cells, osteoblast, osteoporosis, single-cell RNA sequencing, spatial transcriptomics

## Abstract

Bone formation is spatially organized, yet the molecular architecture of the bone formation niche has only recently become accessible. Osteoporosis is a major skeletal disorder characterized by bone loss, microarchitectural deterioration, and increased fracture risk. Although antiresorptive and anabolic therapies are effective, a key conceptual gap remains: the cellular components of bone formation are increasingly well defined, but how these cells are spatially arranged and coordinated in health and disease is less understood. Recent advances in single-cell RNA sequencing and spatial transcriptomics now allow transcriptome-wide mapping of bone-forming microenvironments in intact human and mouse tissues. This review synthesizes emerging findings into a spatial framework for understanding bone formation and osteoporosis. We trace the conceptual lineage from the bone-remodeling compartment canopy hypothesis to its molecular refinement through recent single-cell and spatial atlases. We integrate evidence for mesenchymal stromal cell heterogeneity and spatial niche organization to propose a three-zone model of the bone formation niche, comprising a canopy or marrow-side stromal zone, a reversal or transition zone, and a bone-surface formation zone. We further propose “spatial de-zonation” as a hypothesis-generating framework for osteoporosis, referring to the potential erosion of spatial gradients that normally separate osteogenic, stromal, vascular, and adipogenic domains. Importantly, the available human spatial atlases were generated predominantly from osteoarthritic femoral heads and therefore provide reference maps rather than direct evidence of osteoporosis-specific niche disorganization. Finally, we consider niche-restorative therapeutic strategies that may move beyond targeting individual cell types toward restoring the architectural integrity of the bone formation niche.

## 1 Introduction

Osteoporosis affects over 200 million people worldwide and accounts for approximately 9 million fragility fractures annually (1, 2). The disease is characterized by progressive bone loss and microarchitectural deterioration, resulting from an imbalance between osteoclast-mediated bone resorption and osteoblast-mediated bone formation. Current pharmacotherapy, dominated by antiresorptive agents such as bisphosphonates and denosumab, effectively reduces fracture risk, but does little to restore lost bone mass (3). Anabolic agents, including teriparatide and romosozumab, can stimulate bone formation; however, their efficacy is constrained by treatment duration limits and incomplete recovery of bone microarchitecture (4). This therapeutic gap exposes a fundamental problem: we know which cells participate in bone formation, but we understand remarkably little about where and how they are organized to execute this function.

The classical model of bone remodeling describes a tight spatiotemporal coupling between osteoclasts and osteoblasts within the basic multicellular unit (BMU) (5). However, the cellular and molecular details of how osteoblast precursors are recruited to previously resorbed bone surfaces, how they differentiate *in situ*, and how the entire process is spatially orchestrated have remained elusive. A major conceptual advance came from Andersen, Delaissé, and colleagues, who used transmission electron microscopy and immunohistochemistry to describe the bone remodeling compartment (BRC), a thin cellular “canopy” of mesenchymal origin that physically separates the remodeling site from the marrow cavity and serves as a reservoir of osteoprogenitors (6–8). Critically, they demonstrated that the absence of BRC canopies correlates with arrested reversal phase and deficient bone formation in postmenopausal osteoporosis (6), glucocorticoid-induced osteoporosis (7), and multiple myeloma (9). These observations established a key principle: the spatial organization of the bone surface microenvironment is not a passive structural feature, but an active determinant of bone formation capacity.

Despite this foundational insight, the molecular identity of canopy cells and the full cellular composition of the bone formation niche have remained unknown, largely because tools to profile such spatially restricted microenvironments at transcriptome-wide resolution did not exist. This changed dramatically in 2024–2025 with two parallel breakthroughs. First, scRNA-seq applied to enzymatically released bone cells, rather than bone marrow aspirates, has captured the full spectrum of bone-resident mesenchymal stromal cell (MSC) subtypes, revealing previously hidden populations such as Fibro-MSCs, Osteo-MSCs, and Adipo-MSCs with distinct transcriptional identities and spatial distributions (10). Second, spatial transcriptomics has overcome the formidable technical challenge of profiling heavily mineralized tissue, enabling the direct mapping of gene expression onto bone tissue architecture (11–13). The resulting datasets constitute the first molecular atlases of the intact bone formation microenvironment.

A parallel conceptual advance has been the recognition that skeletal stem cells (SSCs) do not represent a single monolithic cell type, but rather a family of related cells distinguished by distinct anatomic locations and functional specializations. Bok et al. (2024) recently synthesized evidence from multiple laboratories supporting the site specificity of skeletal stem cells. Vertebral Pax1^+^Zic1^+^ SSCs, calvarial CTSK^+^ and DDR2^+^ SSCs, growth plate-resident PTHrP^+^ stem cells, and periosteal and endosteal SSCs each generate osteoblasts with distinct transcriptional and functional features that reflect their anatomical origin (14). This model is supported by experimental evidence: Sun et al. (2023) identified a vertebral skeletal stem cell (vSSC) co-expressing ZIC1 and PAX1 that is distinct from long bone SSCs (15). vSSCs display formal evidence of stemness, self-renewal, label retention, and a position at the apex of their differentiation hierarchy. These cells were also shown to contribute to the preferential metastasis of breast cancer cells to the spine relative to other skeletal sites, in part through secretion of the metastatic trophic factor MFGE8. This finding demonstrates that site-specific SSC identity translates directly into site-specific disease susceptibility, reinforcing the paradigm that osteoblasts are not a single cell type but a family of cells with distinct origins and functions. Additional evidence for this diversity comes from endosteal stem cells (16) and periosteal stem cells, which contribute to distinct bone compartments, and from the finding that aged SSCs lose bone-forming capacity while generating a pro-inflammatory niche (18). Collectively, these conceptual and technological advances set the stage for a fundamental re-examination of the bone formation niche.

In this review, we examine how recent single-cell and spatial technologies are redefining the bone formation niche—the spatially organized microenvironment in which osteoprogenitors are recruited, differentiate, and execute matrix deposition. We trace the conceptual development of the bone remodeling compartment canopy model, synthesize emerging evidence that bone formation is governed by a spatially structured niche composed of skeletal progenitors, osteoblast-lineage cells, endothelial cells, and immune components, and discuss how this framework reframes bone formation as a niche-dependent program rather than a simple linear differentiation process. We further consider how this perspective may inform future therapeutic strategies for osteoporosis.

## 2 The bone remodeling compartment: a historical foundation

**Figure 1:**
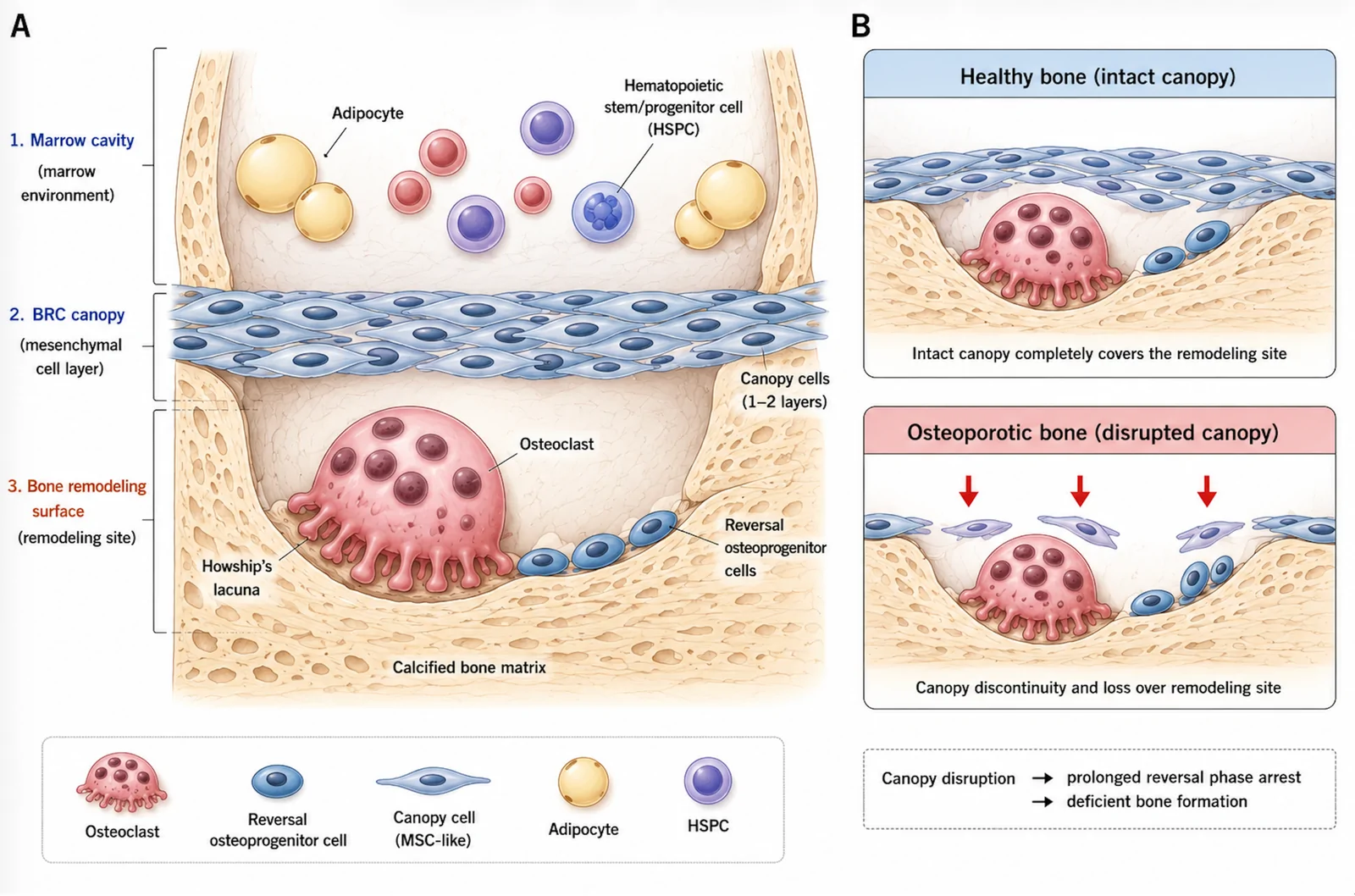
The bone remodeling compartment canopy model and its disruption in osteoporosis. **(a)** Schematic representation of a trabecular bone remodeling site showing the bone marrow cavity, bone remodeling compartment (BRC) canopy, and bone remodeling surface. The BRC canopy is a thin mesenchymal cell layer that separates the remodeling surface from the general marrow environment. Beneath the canopy, osteoclasts resorb bone in Howship’s lacunae, while reversal osteoprogenitor cells accumulate along the eroded surface to support the transition from resorption to bone formation. **(b)** In healthy bone, an intact canopy maintains a protected remodeling microenvironment and supports osteoprogenitor recruitment. In osteoporosis, canopy disruption exposes the remodeling surface to the marrow cavity, contributing to reversal-phase arrest, impaired osteoblast recruitment, and deficient bone formation.

The idea that bone remodeling takes place within a structurally defined and spatially segregated microenvironment arose from detailed ultrastructural studies conducted over the past two decades. Using transmission electron microscopy of human iliac crest biopsies, Andersen and colleagues identified a thin cellular envelope, only one to two cell layers thick, covering active remodeling sites. They termed this structure the bone-remodeling compartment canopy (6, 19). The canopy appeared continuous with bone-lining cells at the periphery of the remodeling surface, thereby forming a semi-enclosed compartment that separates the remodeling site from the surrounding bone marrow (**Figure 1A**).

Functionally, the BRC canopy was proposed to serve three critical roles. First, it may act as a reservoir of osteoprogenitor cells that are delivered to the eroded (reversal) bone surface to initiate the bone formation phase (8). Immunohistochemical analysis revealed that canopy cells express osteoblast lineage markers (alkaline phosphatase, osterix), and that the density of osteoprogenitors on the reversal surface must reach a critical threshold before bone formation can commence. In intracortical remodeling of adolescent human bone, Christiansen et al. (2023) quantified this threshold at approximately 43 osteoprogenitor cells per mm of eroded surface (20), providing the first quantitative evidence that canopy-mediated progenitor delivery is rate-limiting for bone formation. Second, the canopy may create a relatively segregated microenvironment that concentrates local signals (growth factors released from bone matrix during resorption, paracrine factors from reversal cells) necessary for osteoblast differentiation. Third, the canopy physically separates the remodeling site from the marrow cavity, preventing interference from marrow-derived cells during the delicate transition from bone resorption to formation.

The clinical relevance of the BRC canopy was established through histomorphometric studies in osteoporotic patients. In postmenopausal women with osteoporosis, Andersen et al. (2014) found a significant reduction in canopy coverage over remodeling sites compared to age-matched controls, and this reduction was associated with arrest of the reversal phase—the intermediate stage between completion of bone resorption and initiation of bone formation (6). Similarly, in glucocorticoid-induced osteoporosis, Jensen et al. (2015) demonstrated that both canopy cells and the underlying reversal cells are jointly affected, resulting in a compounded defect of progenitor supply and differentiation capacity (7). These findings led to a compelling model: osteoporosis is not merely a quantitative imbalance between resorption and formation, but a qualitative failure of the spatial infrastructure required for bone formation—the BRC canopy is disrupted, osteoprogenitors cannot be adequately recruited to the eroded surface, and the reversal phase stalls (**Figure 1B**).

A key mechanistic insight came from Delaisse et al. (2020), who synthesized histomorphometric data from human cancellous bone into a unified model of the reversal phase (21). This model revealed that osteoclasts and osteoprogenitors are intermingled on eroded surfaces, contrary to the traditional sequential view, and that osteoprogenitor cell expansion proceeds as an integrated mechanism together with resorption. The osteoclast itself was identified as the cell that initiates osteoprogenitor expansion by triggering reservoirs positioned in proximity to the eroded bone surface: bone lining cells (BLCs), canopy cells, and pericytes. Critically, a threshold cell density of osteoprogenitors on the eroded surface is mandatory for the onset of bone formation; if this threshold is not reached, uncoupling and permanent bone loss occur (21). This threshold concept provides a quantitative framework for understanding why canopy disruption leads to bone formation failure: without a structurally intact canopy to deliver progenitors, the critical cell density cannot be achieved. The five-phase bone remodeling cycle—quiescence, activation, resorption, reversal, and formation—and the cellular players involved in each phase have been comprehensively reviewed elsewhere (22). Importantly, the reversal phase is not merely transitional but is an active, regulated process: Borggaard et al. (2022) demonstrated that alendronate, a first-line bisphosphonate, significantly prolongs the reversal-resorption phase in human cortical bone remodeling, directly showing that antiresorptive therapy can alter the spatiotemporal dynamics of the remodeling cycle (17). This work highlights a previously underappreciated way in which antiresorptive agents affect the cellular kinetics of bone remodeling beyond their primary action on osteoclasts.

In 2023, Andersen and colleagues further defined the cellular identity of the canopy by characterizing the bone marrow envelope, a CD271^+^/PDGFRβ^+^ cell layer that lines the marrow-facing bone surface. This envelope was shown to possess osteoblastogenic potential not only at active remodeling sites but also along quiescent bone surfaces (23). At active remodeling sites, the BME assumes a canopy-like morphology and exhibits early osteoblastogenic activation. This is reflected by increased expression of Ki67, smooth muscle actin (SMA), tenascin C (TNC), fibronectin (FN1), and MMP13, which appear concurrently with the initiation of bone remodeling. Importantly, the high proliferation index in the canopy was not associated with increasing cell density at the canopy level, but rather at the bone surface level, consistent with a model of active cell delivery from the canopy to the bone surface (23). In quiescent bone, CD271 and PDGFRβ were detected in more than 90% of the cells covering bone surfaces, indicating that the entire bone surface is invested with osteoblastogenic potential—a latent infrastructure poised to respond to remodeling signals. This work supported the interpretation of the BME/canopy as a continuous functional unit that integrates osteoblastogenesis and bone remodeling into a single spatially coordinated process (21, 23).

Despite its conceptual importance, the BRC canopy model has several limitations. First, much of the supporting evidence derives from two-dimensional histological and immunohistochemical observations of human iliac crest or cancellous bone specimens (6–8, 19, 21, 23). These studies demonstrate associations among canopy coverage, osteoprogenitor density, reversal-phase progression, and bone formation, but do not by themselves establish causality. Second, the canopy is a thin and potentially discontinuous three-dimensional structure, and its apparent presence or absence may be influenced by section orientation, sampling location, tissue processing, and marker selection. Third, its cellular boundaries and molecular identity remain incompletely resolved, and its relationship to bone-lining cells, bone marrow envelope cells, pericytes, and adjacent stromal progenitors has not been fully defined (21, 23).

Most importantly, robust functional validation in genetically tractable murine models remains limited. Direct testing would require selective labeling, perturbation, or ablation of canopy-associated cells, followed by longitudinal assessment of osteoprogenitor recruitment, the reversal-to-formation transition, and bone formation. However, a murine structure anatomically and molecularly equivalent to the human BRC canopy has not yet been definitively established. Species differences in trabecular organization, remodeling patterns, and the prevalence of intracortical remodeling further complicate translation. The canopy model should therefore be regarded as a strongly supported human histological framework whose causal mechanisms and cross-species conservation remain to be experimentally demonstrated (21, 23).

These limitations also highlight several unresolved molecular questions. The transcriptional programs that define canopy-associated cells, the signals that direct their assembly and maintenance, and their molecular communication with osteoclasts and reversal cells remain unclear. Addressing these questions requires technologies capable of profiling spatially restricted cell populations at transcriptome-wide resolution. Recent advances in single-cell and spatial transcriptomics have begun to provide these capabilities, as discussed in the following section.

## 3 Technological foundations: single-cell and spatial transcriptomics enter mineralized tissue

### 3.1 The challenge of mineralized tissue

Bone presents unique challenges for transcriptomic profiling. The hydroxyapatite mineral matrix must be removed to release cells (for scRNA-seq) or to section the tissue (for spatial transcriptomics), yet the decalcification process itself can severely compromise RNA integrity. Traditional decalcification using ethylenediaminetetraacetic acid (EDTA) requires days to weeks, during which endogenous RNases degrade mRNA. For spatial transcriptomics, the consequences have been severe: early attempts to apply 10X Visium to bone tissue detected as few as 172–310 genes per 55-μm spot—an order of magnitude lower than the 2,000–4,000 genes routinely obtained from soft tissues (11). Lin et al. (2025) obtained an average of 264 genes per spot across 2,947 spots from femoral head tissue after EDTA decalcification (12), illustrating the difficulty even with optimized protocols. This limitation has been a major bottleneck for spatial transcriptomics in bone research.

A methodological breakthrough came in 2025 from Miao and colleagues, who developed a practical protocol replacing EDTA with Morse’s solution (22.5% formic acid with 10% sodium citrate) for rapid decalcification of mineralized tissue (24). This approach achieves complete decalcification in less than 24 hours while preserving RNA integrity (DV200 values of 50–60%). When applied to 10X Visium V2, the protocol yielded an average of 5,639 genes per spot—a >20-fold improvement over conventional EDTA-based methods. For the higher-resolution Visium HD platform, approximately 170 genes were detected per 8-μm bin (equivalent to ~4,100 genes per 55-μm spot). Miao et al. also introduced the use of SCHOTT NEXTERION Slide H, a 3-D hydrogel-coated slide that prevents detachment of fragile decalcified bone sections. This protocol represents a step-change in the feasibility of bone spatial transcriptomics, although it has yet to be widely adopted in published bone atlases.

### 3.2 Platforms for spatial transcriptomics in bone

The current spatial transcriptomics landscape relevant to bone research divides into sequencing-based and imaging-based methods.

Sequencing-based approaches, exemplified by 10X Visium (V2 and HD), capture the full transcriptome from tissue sections placed on spatially barcoded slides. Visium CytAssist is compatible with formalin-fixed paraffin-embedded (FFPE) samples as used by Lin et al. (2025) for their human femoral head atlas (12), expanding applicability to archival clinical bone specimens. However, Visium’s spatial resolution (55 μm for V2 spots, 2 μm bins for HD) remains a significant limitation for studying the BRC canopy, which is only 10–30 μm thick. A single Visium V2 spot would encompass the entire canopy plus underlying marrow cells, making it impossible to resolve the canopy as an isolated structure. This resolution constraint means that the cellular composition inferred for canopy-proximal regions is necessarily a mixture of canopy cells and cells in the immediately adjacent marrow. A comprehensive review by Jenkins et al. (2025) of spatial transcriptomic applications across orthopedic tissues found that sequencing-based methods (10X Visium) have been used in 22 of 29 published studies, reflecting their current dominance, while imaging-based methods are increasingly recognized as superior for hypothesis-driven investigation at subcellular resolution (25).

Imaging-based methods offer superior spatial resolution at the cost of transcriptome coverage. CODEX (CO-Detection by indEXing), used by Bandyopadhyay et al. (2024) to map 53 proteins across >1.2 million cells in human bone marrow, achieves subcellular resolution but is limited to preselected antibody panels (10). MERFISH and 10X Xenium can profile hundreds to thousands of genes at subcellular resolution and are likely to become the methods of choice for resolving thin structures such as the BRC canopy once bone-specific gene panels are designed. For now, the field relies on a complementary strategy: scRNA-seq to comprehensively characterize cellular heterogeneity, Visium to provide tissue-scale spatial context, and CODEX/immunofluorescence to validate key findings at cellular resolution. This integrative approach underpins all of the major discoveries discussed in this review (10, 12, 26).

### 3.3 scRNA-seq: capturing the full cellular repertoire of bone

A critical methodological insight from the past two years is that the method of cell isolation fundamentally determines which bone-resident cell populations are captured. Bandyopadhyay et al. (2024) demonstrated that enzymatic digestion of femoral head tissue, rather than bone marrow aspiration, yields approximately 24.4-fold more non-Adipo-MSC subsets compared with all prior adult scRNA-seq datasets combined (10). Crucially, Fibro-MSCs, which emerged as the most primitive MSC subset with the highest CFU-F capacity, were essentially absent from prior adult datasets obtained by marrow aspiration. This finding underscores a general principle: matrix-adherent populations, potentially including canopy cells and bone-lining cells, are underrepresented or missed by aspiration, and their transcriptional identities remained largely hidden until the advent of enzymatic release protocols. The recent integrative analysis by Zhou et al. (2025), which pooled 77 scRNA-seq libraries from 14 independent datasets to analyze approximately 181,000 non-hematopoietic bone marrow stromal cells, further demonstrates the power of large-scale data integration for identifying rare and compartment-restricted cell populations (27).

Together, these methodological advances provide the technical basis for reconstructing the bone formation niche at cellular and spatial resolution. Single-cell RNA sequencing enables systematic profiling of cellular heterogeneity in bone tissue, especially when optimized digestion protocols recover matrix-adherent mesenchymal stromal cells and osteolineage populations that are underrepresented in conventional marrow aspirates. However, dissociation-based approaches inevitably lose anatomical context. Spatial transcriptomic platforms, particularly when combined with bone-compatible decalcification protocols such as Morse’s solution-based rapid decalcification, preserve tissue architecture and allow gene expression to be mapped onto histological bone sections. Because spot-based spatial transcriptomics does not always resolve individual cell identities, integration with single-cell or single-nucleus reference datasets, multiplex imaging, and histomorphometric information is essential for accurate niche reconstruction. This multi-platform strategy enables the construction of spatially annotated bone cell atlases and provides a framework for linking cellular states, molecular gradients, and anatomical niche organization in bone formation and osteoporosis.

The key studies that have established the technological and biological foundation for spatially resolved bone research, together with their platforms, sample types, principal findings, and interpretive limitations, are summarized in **Table 1**. However, molecular atlases alone cannot determine whether the matrix produced within a spatially defined niche has appropriate composition and mechanical competence. Complementary biophysical approaches are therefore required to connect niche organization with bone quality.

**Table 1:** Representative single-cell, spatial, histological, and biophysical studies informing the bone formation niche framework.

Study	Approach/platform	Sample/model	Key contribution to the spatial niche framework	Disease/site context and interpretive limitation	Ref
Lin et al., 2025	10X Visium CytAssist + scRNA-seq	Human femoral head, osteoarthritis	Generated an adult human bone spatial atlas; identified a bone surface-associated osteogenic niche enriched in COL1A1, SPP1, SPARC, and RUNX2^+^ osteoblastic-lineage cells	OA femoral head; not an OP cohort. The spatial atlas was derived from a single donor, and Visium spot resolution is insufficient to resolve the thin BRC canopy or inter-individual variability.	(12)
Bandyopadhyay et al., 2024	scRNA-seq + 53-plex CODEX imaging	Human femoral heads from 12 OA patients undergoing total hip arthroplasty (52–74 years; CODEX: 12 specimens, including 8 transcriptomic samples; sex distribution not reported)	Defined spatially segregated MSC subsets, including Fibro-MSCs, Osteo-MSCs, and Adipo-MSCs; mapped Osteo-MSCs near trabecular bone and Adipo-MSCs deeper in marrow	Arthroplasty/OA femoral heads; 12 patients, 52–74 years (reported age summary 65 ± 8 years); sex distribution not reported in the main article. CODEX uses a preselected antibody panel and 2D sections; these data cannot demonstrate OP-specific spatial changes.	(10)
To et al., 2024	snRNA-seq + snATAC-seq + ISS + Visium	Human embryonic skeleton	Identified a spatial molecular switch at the osteogenic front, linking TWIST1-associated non-ossifying mesenchyme to RUNX2/SP7-driven osteogenesis	Developmental human skeleton rather than adult or osteoporotic bone; developmental-stage and skeletal-site context limits extrapolation to adult fragility-fracture niches.	(26)
Zhou et al., 2025	Integrative scRNA-seq meta-analysis	Mouse bone and marrow stromal datasets	Defined compartment-related osteoblast-lineage populations, including marrow/endosteal OLC1 and periosteal OLC2, with distinct transcriptional regulons and ageing vulnerability	Integrative mouse datasets provide cross-study cellular evidence but no direct human OP spatial validation; species, skeletal-site, age, and protocol heterogeneity limit translation.	(27)
Liao et al., 2025	scRNA-seq + lineage tracing + intersectional genetics	Adult mouse bone marrow	Identified endosteal Adipoq^+^Osx^+^ bipotent progenitors that generate osteoblasts and marrow adipocytes; showed fate shift toward adipogenesis under estrogen deficiency	Functional lineage tracing and genetic perturbation strengthen causality, but the findings derive from mouse endosteal marrow/estrogen-deficiency models; species and skeletal-site differences remain.	(29)
Deng et al., 2025	10X Visium	Mouse femur and adjacent muscle	Mapped spatial ligand–receptor communication across bone and muscle, including COLLAGEN, THBS, VEGF, FN1, and TENASCIN signaling pathways	Mouse femur and adjacent muscle rather than human OP bone; spot-level signals may mix multiple cell types, and cross-species/site translation is uncertain.	(32)
Miao et al., 2025	10X Visium V2/HD protocol optimization	Mouse bone	Established a bone-compatible spatial transcriptomic workflow using Morse’s solution, markedly improving RNA preservation and gene detection compared with EDTA	Mouse protocol-optimization study; establishes technical feasibility but does not validate disease-specific niche biology in human OP.	(24)
Noom et al., 2025	scRNA-seq + MELC imaging	Mouse fracture callus, young versus aged	Identified Spp1^hi macrophages as spatially restricted osteoclast precursors and showed that ageing disrupts cortical niche coordination during repair	Mouse fracture callus represents repair rather than steady-state OP remodeling; species, age, and anatomical-site differences constrain generalization.	(36)
Huang et al., 2024	scRNA-seq + bulk RNA-seq + mouse genetics	Mouse bone marrow under estrogen deficiency/obesity	Identified ESRRA as an adipocyte-mediated regulator of the adipo-osteogenic switch; ESRRA inhibition preserved osteogenesis and type H vessels	Mouse genetics provide functional evidence under estrogen deficiency/obesity, but spatial de-zonation is inferred rather than directly mapped; species and site differences remain.	(35)
Delaissé et al., 2020	Histomorphometric synthesis	Human cancellous bone	Proposed a unified reversal-phase model in which threshold osteoprogenitor density on eroded surfaces is required for initiation of bone formation	Human cancellous-bone evidence is anatomically relevant, but the model is derived mainly from 2D histomorphometric associations and lacks selective functional perturbation.	(21)
Andersen et al., 2014; Jensen et al., 2015; Andersen et al., 2023	IHC/histomorphometry	Human iliac crest/cancellous bone; postmenopausal OP, glucocorticoid-induced OP, and reference samples	Linked reduced canopy coverage to reversal-phase arrest and deficient bone formation; characterized the CD271^+^/PDGFRβ^+^ bone marrow envelope as an osteoblastogenic canopy-associated layer	Related human iliac-crest studies directly associate canopy loss with postmenopausal and glucocorticoid-induced OP, but the evidence remains 2D, histological, and correlative; robust murine functional validation is lacking.	(6, 7, 23)
Morris and Mandair 2011; Unal et al., 2021; Shah 2020	Raman spectroscopy; methodological and compositional assessment	Human and animal bone literature; human cortical-bone measurement examples	Established Raman-derived measures of mineral and matrix composition, including mineral-to-matrix and carbonate-to-phosphate ratios, crystallinity, and collagen-associated spectral features	Provides compositional rather than cellular information; measurements are sensitive to fluorescence, preparation, preprocessing, and peak selection. Not spatially co-registered with an OP niche atlas.	(41–43)
Cardinali et al., 2019; Alunni Cardinali et al., 2021	Brillouin micro-spectroscopy; combined Brillouin–Raman mapping	Ex vivo human femoral head/epiphysis; cortical and trabecular bone	Mapped microscale mechanical heterogeneity and enabled co-localized chemical and micromechanical characterization of bone compartments	Ex vivo, anatomically restricted, and based on limited donor material; Brillouin signals depend on hydration, refractive index, density, and anisotropy. No OP-specific spatial-omics co-registration.	(44, 45)

### 3.4 Integrating spatial omics with the material properties of bone

Bone should be considered not only as a cellular tissue but also as a hierarchical biomaterial. Spatial transcriptomics can identify the cells and signaling programs associated with matrix formation, but transcript abundance does not directly determine the composition or mechanical competence of the matrix ultimately produced. This distinction is particularly important in osteoporosis, in which fracture risk reflects not only bone quantity but also mineral composition, collagen organization, microdamage, and tissue-level mechanical heterogeneity. Integrating molecular and biophysical measurements may therefore help connect spatial niche organization to bone quality and fracture resistance.

Raman spectroscopy provides label-free information on the chemical composition of bone, including mineral-to-matrix ratio, carbonate-to-phosphate ratio, mineral crystallinity or maturity, and collagen-associated spectral features (41, 42). Spatial Raman mapping could therefore determine whether osteogenic territories identified by transcriptomic methods correspond to local differences in matrix composition and maturation. Such measurements may help distinguish a transcriptionally active osteogenic niche from one that produces compositionally abnormal or mechanically inferior matrix. Raman-based measurements have already been used to characterize variations in bone composition associated with aging, osteoporosis, fracture history, and therapeutic intervention, supporting their potential value as a bridge between molecular state and tissue quality (41–43).

Brillouin light-scattering microscopy provides complementary, label-free information on local longitudinal mechanical properties and viscoelastic heterogeneity without requiring physical contact with the sample (44). When combined with Raman spectroscopy, it enables co-localized chemical and micromechanical characterization of cortical and trabecular bone (45). Integration of spatial transcriptomics, histology, Raman mapping, and Brillouin microscopy could therefore link cellular state and niche organization to the composition and mechanics of newly formed bone. For example, regions showing canopy disruption or depletion of osteoblast-lineage programs could be examined for altered mineral-to-matrix ratio, collagen organization, mineral maturity, or local longitudinal modulus. At present, however, this proposed relationship remains to be directly demonstrated in osteoporotic human bone.

These approaches also have important limitations. Raman measurements may be influenced by tissue fluorescence, tissue processing, spectral preprocessing, instrument configuration, and the choice of spectral bands or peak ratios (42, 43). Brillouin measurements are affected by hydration, refractive index, mass density, and structural anisotropy, and the Brillouin-derived longitudinal modulus should not be interpreted as directly equivalent to the Young’s modulus obtained from conventional mechanical testing (44, 45). Meaningful integration with spatial omics will therefore require standardized specimen preparation, rigorous spatial co-registration, and validation against established compositional and mechanical measurements.

## 4 Cellular building blocks of the spatial bone formation niche

### 4.1 MSC heterogeneity: multiple osteoprogenitor populations

**Figure 2:**
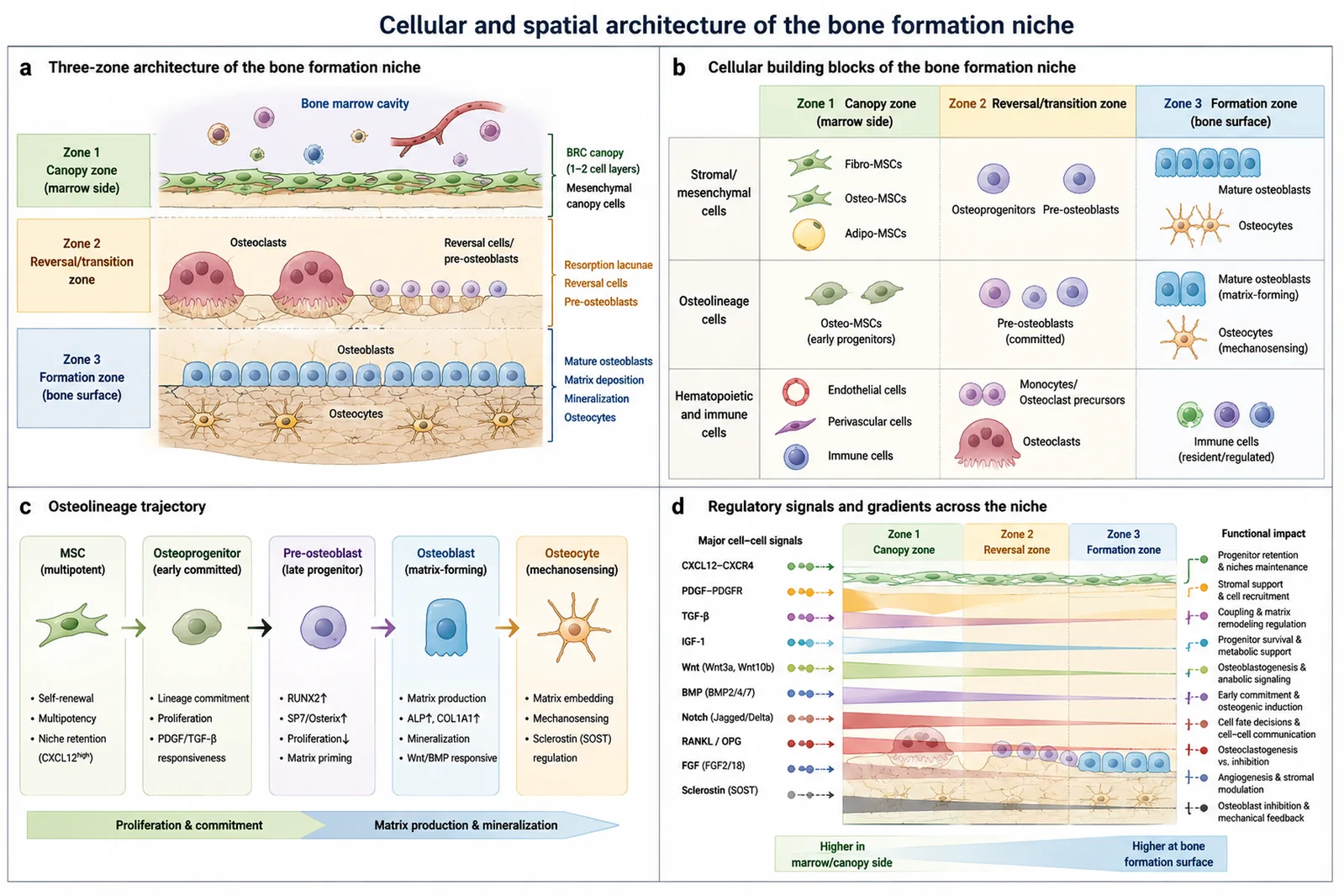
Cellular and spatial architecture of the bone formation niche. **(a)** Three-zone model of the bone formation niche surrounding a trabecular remodeling surface. The marrow-side canopy zone contains mesenchymal canopy cells; the reversal/transition zone contains osteoclasts, reversal cells, and pre-osteoblasts; and the bone-surface formation zone contains matrix-forming osteoblasts and embedded osteocytes. **(b)** Major cellular components within each zone, including stromal/mesenchymal populations, osteolineage cells, endothelial and perivascular cells, immune cells, and osteoclast-lineage cells. **(c)** Osteolineage progression from mesenchymal stromal cells to osteoprogenitors, pre-osteoblasts, mature osteoblasts, and osteocytes, accompanied by increasing matrix production, mineralization, and mechanosensing functions. **(d)** Representative cell–cell signals and molecular gradients coordinating niche organization, osteoprogenitor recruitment, osteoblast differentiation, remodeling coupling, and osteocyte-mediated feedback.

The most comprehensive dissection of human bone MSC heterogeneity to date comes from Bandyopadhyay et al. (2024), who profiled 29,325 non-hematopoietic cells from enzymatically digested femoral heads and identified nine transcriptionally distinct subtypes (10). Among these, four populations are directly relevant to bone formation. (1) Osteo-MSCs and mature osteoblasts, characterized by NCAM1, SPP1, IBSP, and BGLAP expression, represent the committed osteogenic lineage. (2) Adipo-MSCs, marked by APOE, LPL, PPARG, and the highest CXCL12 expression among all MSC subsets, are the dominant source of the HSPC niche factor CXCL12. (3) THY1+ MSCs are an adipocytic-skewed population with no clear murine counterpart. (4) Fibro-MSCs, defined by podoplanin (PDPN), DCN, DPT, and the highest CFU-F capacity, represent the most primitive, stem-like MSC population and align most closely with the ISCT criteria for MSCs (10). CODEX-based 53-plex spatial proteomic imaging of more than 1.2 million cells further showed that MSC subtypes are spatially segregated within the marrow. Osteo-MSCs were enriched near trabecular bone surfaces, consistent with a canopy-like niche, whereas Adipo-MSCs localized deeper in the marrow cavity near adipocytes, supporting a spatially compartmentalized model of the bone formation niche (**Figure 2**). Notably, CODEX data also revealed that adult hematopoietic stem and progenitor cells (HSPCs) are predominantly peri-adipocytic, challenging the historical emphasis on an exclusively endosteal HSPC niche (10).

The concept of MSC spatial heterogeneity is further supported by the discovery of anatomically restricted skeletal stem cell populations. Matsushita et al. (2023) identified endosteal stem cells that are molecularly and functionally distinct from both periosteal and central marrow SSCs, and demonstrated that these cells dictate active osteogenesis on endosteal bone surfaces (16). Complementing this, Ambrosi et al. (2021) showed that aged SSCs lose their bone-forming capacity and instead generate a pro-inflammatory, degenerative niche, providing a direct mechanistic link between stem cell aging and the deterioration of the bone formation microenvironment (18). Together, these findings establish that skeletal stem and progenitor cells are not uniformly distributed throughout the marrow but are spatially compartmentalized, with each compartment contributing differentially to bone formation, hematopoiesis, and, when dysregulated, to skeletal pathology.

### 4.2 Two distinct osteolineage populations: OLC1 and OLC2

Complementary work by Zhou et al. (2025) focused specifically on osteolineage cells (OLCs) by integrating 77 scRNA-seq libraries from 14 independent datasets, analyzing ~181,000 non-hematopoietic stromal cells from mouse bone marrow (27). This meta-analysis identified two distinct OLC populations with fundamentally different biological properties. OLC1 cells (17,137 in adult mouse bone marrow) derive from Leptin receptor-positive (Lepr^+^) bone marrow MSCs and are characterized by expression of osteoblast differentiation genes including Gdpd2, Satb2, Id4, and Wnt4. OLC1 localizes predominantly to the bone marrow and endosteal surface. OLC2 cells (6,835), in contrast, originate from Gli1^+^ periosteal MSCs (P-MSCs) and are specialized for extracellular matrix (ECM) organization, uniquely expressing Nppc, Ostn, Tnn, and Sfrp2. OLC2 resides mainly in the bone periosteum, distinguishing it from the marrow-derived OLC1 trajectory. Both populations can be isolated as PDGFRβ^+^NCAM1^+^ cells; OLC1 is Bst2^+^ while OLC2 is Bst2^−^, providing a practical means of prospective isolation (27).

The transcriptional regulation of these two lineages is governed by distinct sets of transcription factors. SCENIC analysis revealed that OLC1 development is driven by seven regulatory modules, with Sp7 (osterix), Mef2c, Creb3l1, Dlx3, Tbx2, Zfhx3, and Klf4 as the key regulators—six of which are directly involved in osteoblast differentiation and skeletal development (27). RNA velocity analysis further identified Sp7, Mef2c, Creb3l1, and Satb2 as the master drivers whose expression positively correlates with OLC1 lineage progression. In contrast, OLC2 development is controlled by 16 regulons, with Prrx2, Maf, Shox2, Dlx5, Jun, and Tbx18 as the core drivers, 13 of which are implicated in skeletal system development and ossification (27). This fundamentally different upstream regulatory logic means that bone marrow-derived and periosteum-derived osteogenesis are controlled by distinct transcriptional programs, with important implications for understanding site-specific bone loss in osteoporosis.

Both OLC1 and OLC2 showed significant polygenic association with bone mineral density (BMD) by single-cell disease relevance score (scDRS) analysis, with the association increasing along the differentiation trajectory and osteoblasts showing the highest relevance score, consistent with the notion that mature bone-forming cells, rather than progenitors, are the primary effectors of BMD genetics (27). Notably, the BMD-relevant genes differed between the two populations: OLC1-associated genes (n = 249) were enriched for skeletal development functions (Bmp1, Bmp3, Satb2, Dmp1), while OLC2-associated genes (n = 119) were predominantly involved in ECM organization (Col1a1, Col1a2, Col11a1, Postn, Fn1). Importantly, OLC2-specific ECM proteins were selectively decreased in bone tissue of aged mice, and both OLC1 and OLC2 were consistently reduced in aged animals. The authors propose that decreased expression of lineage-specific transcription factors, Sp7/Mef2c/Creb3l1 for OLC1 and Prrx2/Maf/Shox2 for OLC2, may underlie this age-related depletion (27).

### 4.3 Osteostaticytes: the signal that couples resorption to formation

A missing link in the BRC canopy model has been the identity of the molecular signal that attracts osteoprogenitors to the reversal surface after osteoclasts complete resorption. A striking discovery by Wei et al. (2024) identified a novel osteoclast-lineage subset termed “osteostaticytes” (OSCs), defined by high expression of IDO1, CCL3, and CCL4 (28). scRNA-seq of human cortical bone revealed that OSCs represent an early stage of the osteoclast lineage and exhibit the highest chemotactic activity toward MSCs among all osteoclast-lineage subsets tested. Functionally, OSCs are proposed to be the long-sought “reversal cells” that occupy eroded bone surfaces after osteoclasts have departed and secrete chemokines to recruit osteoblast precursors from the canopy and surrounding marrow. This discovery provides a molecular mechanism for the coupling of bone resorption to formation at the level of cell recruitment—the very process that the BRC canopy spatially facilitates. Whether OSCs themselves reside within the canopy-covered compartment and how their chemokine secretion is spatially directed remain important open questions amenable to spatial transcriptomic investigation.

### 4.4 Adipo-osteoprogenitors in the endosteal niche

Adding further complexity to the osteoprogenitor landscape, Liao et al. (2025) provided a detailed characterization of adipo-osteoprogenitor cells in adult mouse bone marrow (29). By performing scRNA-seq of bone marrow stromal cells, the authors identified 13 mesenchymal clusters, among which cluster 3 represented a distinct population of Adipoq^+^Osx^+^ cells. Unlike most Cxcl12-abundant reticular cells, also referred to as marrow adipogenic lineage progenitors, these cells co-expressed adipogenic genes, including Adipoq and Ebf3, together with osteoblast-lineage genes, including Sp7/Osx and Alpl. They also expressed relatively lower levels of Cxcl12, suggesting that they are transcriptionally distinct from conventional CAR/MALP populations.

To confirm that Adipoq and Osx were expressed within the same cells rather than by different cells within the same transcriptional cluster, the authors performed scRNA-seq on FACS-sorted tdTomato^+^ cells from the endosteal fraction of Adipoq-CreERT2;Ai9 mice. Adipoq^+^Osx^+^ cells accounted for 14.57% and 10.45% of Adipoq-lineage mesenchymal cells at 24 hours and 4 weeks after tamoxifen labeling, respectively. Gene set enrichment analysis further showed that oxidative phosphorylation and ribosomal pathways were among the most enriched programs in Adipoq^+^Osx^+^ cells compared with other CAR cells, indicating a more metabolically active state (29).

The spatial localization of Adipoq^+^Osx^+^ cells was established using complementary approaches. In quadruple heterozygous mice carrying Adipoq-CreERT2, Ai9, Osx-rtTA, and tetO-GFP alleles, Adipoq^+^Osx^+^ cells were found predominantly near trabecular and endocortical bone surfaces, whereas double-positive cells were largely absent from the central marrow. Flow cytometry confirmed their enrichment in the endosteal compartment, where Adipoq^+^Osx^+^ cells were more than tenfold more abundant than in the central marrow fraction, accounting for 0.27 ± 0.07% versus 0.02 ± 0.02% of stromal cells, respectively (P < 0.001). These findings define Adipoq^+^Osx^+^ cells as a rare but spatially restricted endosteal progenitor population.

Notably, this progenitor pool exhibited both sexual dimorphism and age-related decline. Among endosteal stromal cells, the proportion of Adipoq^+^Osx^+^ cells was more than twice as high in young females as in young males, but decreased significantly by 12 months of age in both sexes (29). This pattern suggests that depletion of endosteal bipotent progenitors may contribute to age-related impairment of bone formation, particularly in the context of skeletal aging and osteoporosis.

Lineage-tracing and functional studies further established the bipotent nature of Adipoq^+^Osx^+^ cells. In Adipoq-CreERT2;Ai9;Col1(2.3kb)-GFP mice, Adipoq-lineage cells contributed to endosteal osteoblasts, as shown by extensive overlap between tdTomato^+^ Adipoq-lineage cells and GFP^+^ mature osteoblasts after 4 weeks of chase. Conversely, in Osx-CreERT2;Ai9 mice, Osx^+^ progenitors gave rise to marrow adipocytes, particularly under estrogen-deficient conditions. Ovariectomy increased the proportion of tdTomato^+^ adipocytes from 5% in sham-operated mice to 30% in OVX mice, indicating that *de novo* marrow adipogenesis from Osx^+^ progenitors is markedly accelerated by estrogen deficiency (29). This finding provides a cellular explanation for the parallel increase in bone loss and marrow adiposity after menopause.

The most direct evidence for bipotency came from intersectional genetic labeling. Using a novel Sp7-DreERT2 knock-in mouse strain crossed with Adipoq-CreERT2 and the dual-recombinase RC::RLTG reporter, the authors selectively labeled cells with a dual history of Adipoq and Osx expression. At 24 hours after labeling, these dual-recombined cells were rarely found on trabecular bone surfaces and were absent from osteocytes, but were positioned near trabecular and endocortical surfaces, consistent with a progenitor identity. After 4 weeks of chase, dual-labeled cells gave rise to both osteoblasts and marrow adipocytes, providing direct *in vivo* evidence that Adipoq^+^Osx^+^ cells function as bipotent adipo-osteoprogenitors during adult bone marrow homeostasis (29).

Functionally, Osx deletion in Adipoq^+^ cells further demonstrated the importance of this population for trabecular bone formation. Adipoq-Cre: Osx^f/f^ mice developed significant trabecular bone loss, driven by reduced bone formation rather than increased bone resorption, as CTX-I levels were unchanged. In contrast, cortical bone parameters were largely preserved. This dissociation between trabecular and cortical phenotypes suggests that distinct progenitor pools may serve different skeletal compartments. Together, these findings identify Adipoq^+^Osx^+^ cells as a spatially restricted, metabolically active, and functionally bipotent endosteal progenitor population. Their depletion with age and diversion toward adipogenesis under estrogen-deficient conditions provide a potential cellular mechanism linking endosteal niche dysfunction, marrow adiposity, and impaired bone formation in osteoporosis.

Together, the findings discussed above indicate that the bone formation niche is not composed of a single osteoprogenitor population, but rather a spatially organized cellular ecosystem. Distinct MSC subsets, canopy-associated progenitors, endosteal adipo-osteoprogenitors, osteoblast-lineage cells, osteocytes, osteoclasts, and reversal-phase signaling cells occupy defined anatomical positions and perform complementary functions. These cellular constituents are summarized in **Table 2** and integrated into the spatial niche framework shown in **Figure 2**.

**Table 2:** Spatially organized cellular constituents of the bone formation niche.

Cell type	Key markers	Niche location	Putative function	Ref
Fibro-MSC	PDPN, DCN, DPT, CD73, CD105	Zone 1/canopy-marrow-side stromal zone	Primitive stromal MSC; ECM support; progenitor reservoir maintenance	(10)
Osteo-MSC	NCAM1, SPP1, IBSP, RUNX2	Zone 1–2/canopy-proximal and reversal-associated osteogenic region	Osteoblast-lineage commitment; osteoprogenitor supply	(10)
Adipo-MSC	APOE, LPL, PPARG, CXCL12	Marrow-side adipogenic compartment, adjacent to Zone 1	Adipogenic differentiation; CXCL12 source; potential contributor to adipogenic encroachment	(10)
Adipoq^+^Osx^+^ progenitor	Adipoq, Sp7/Osx, Alpl, Ebf3	Endosteal niche/Zone 2–3 interface	Bipotent adipo-osteoprogenitor;osteogenic or adipogenic fate switching	(29)
BME/canopy cell	CD271, PDGFRβ, Ki67, SMA, TNC, FN1, MMP13	Zone 1/BRC canopy	Osteoprogenitor reservoir; niche segregation;progenitor delivery	(23)
Mature osteoblast	RUNX2, COL1A1, SPP1, BGLAP, ALPL	Zone 3/bone-surface formation zone	Matrix deposition and mineralization	(10, 12, 27)
Osteostaticyte	IDO1, CCL3, CCL4	Zone 2/Reversal surface	MSC chemotaxis; reversal coupling	(28)
Osteocyte	SOST, DMP1, FGF23, PHEX	Zone 3/Mineralized bone matrix and lacunae	Mechanosensing; sclerostin secretion;feedback regulation of osteoblast activity	(31)
Osteoclast	ACP5, CTSK, MMP9, CALCR	Zone 2/Resorption lacunae	Bone resorption; remodeling initiation; coupling signal release	(21)

Zone 1 refers to the canopy or marrow-side stromal zone; Zone 2 refers to the reversal or transition zone; and Zone 3 refers to the bone-surface formation zone. These zones represent functional spatial domains rather than rigid anatomical boundaries.

## 5 The bone formation niche in spatial context

### 5.1 Adult human bone atlas and the three-zone spatial model

Recent spatial transcriptomic studies have begun to transform the bone formation niche from an inferred anatomical concept into a molecularly resolved spatial structure. Lin et al. (2025) provided an integrative spatial and single-cell transcriptomic map of adult human bone and bone marrow using 10X Visium CytAssist on FFPE femoral head samples, together with matched scRNA-seq data (12). Despite the technical challenges of transcriptomic profiling in mineralized tissues, this study identified spatially distinct molecular niches within human trabecular bone.

The most relevant finding for bone formation was the identification of a bone surface-associated niche enriched for genes involved in collagen synthesis, matrix organization, bone remodeling, and mineralization, including COL1A1, SPP1, MMP9, and SPARC. Pathway analysis further showed enrichment of mesenchymal differentiation, bone remodeling, and bone mineralization programs in this niche, whereas immune-related pathways characterized the more marrow-associated niche (12). Multimodal integration with scRNA-seq revealed that this osteogenic niche was enriched in mesenchymal lineage cells, particularly RUNX2^+^ osteoblastic-lineage cells and VIM^+^/RUNX2^−^ fibroblasts. These two populations were found in close spatial proximity, suggesting that fibroblast-like stromal cells may participate in shaping the local microenvironment that supports osteoblast differentiation and migration.

A particularly important observation was the spatial restriction of RUNX2 expression. Spots with high RUNX2 expression were concentrated adjacent to trabecular bone surfaces, providing molecular evidence that osteogenic transcriptional activity is spatially confined to the bone surface microenvironment (12). Spatial modeling further identified TGFβ signaling as broadly colocalized with fibroblasts, endothelial cells, and osteoblastic-lineage cells, suggesting that TGFβ may function as a pervasive organizing signal within the bone formation niche. In contrast, NFκB and TNFα signaling showed spatially overlapping distributions, indicating that inflammatory signaling is also embedded within the niche architecture and may become relevant under osteoporotic or inflammatory conditions.

Together with CODEX-based spatial proteomic data showing the segregation of Osteo-MSCs near trabecular bone surfaces and Adipo-MSCs deeper within the marrow cavity (10), these findings support a three-zone model of the bone formation niche (**Figure 2**). In this model, Zone 1 represents the canopy or marrow-side stromal zone, which contains canopy-associated mesenchymal cells, Fibro-MSCs, Osteo-MSCs, vascular and perivascular components, and stromal signals that help maintain progenitor reservoirs and niche segregation. Zone 2 corresponds to the reversal or transition zone, where osteoclast-mediated resorption, reversal cells, osteoprogenitor recruitment, and pre-osteoblast commitment are spatially coordinated. Zone 3 represents the bone-surface formation zone, enriched in mature osteoblasts, matrix deposition and mineralization programs, and embedded osteocytes that provide mechanical and endocrine feedback.

These zones should be understood as functional spatial domains rather than rigid anatomical boundaries. Their biological significance lies in the preservation of gradients between stromal, osteoclastic, osteogenic, vascular, and adipogenic territories. Efficient bone formation may depend not only on the abundance of osteoprogenitors, but also on their spatial alignment with the canopy, reversal surface, vascular support, osteoclast-derived coupling signals, osteocytes, and the mineralized bone matrix. Conversely, erosion of these gradients may represent a central feature of the osteoporotic bone microenvironment and provides the conceptual basis for the spatial de-zonation model discussed below.

### 5.2 Lessons from development: the osteogenic front

The spatial organization of the adult bone formation niche is foreshadowed by the logic of embryonic skeletal development. In a multi-omic atlas of human embryonic skeletal tissues, To et al. (2024) combined single-nucleus transcriptomics, chromatin accessibility profiling, *in situ* sequencing, and spatial transcriptomics to map the emergence of osteogenic domains during development (26). Although this study focused on embryonic skeletal patterning, several of its findings provide a useful conceptual framework for understanding adult bone formation.

A central discovery was the identification of a spatial molecular switch at the osteogenic front of cranial sutures. In the developing suture, mesenchymal populations marked by TWIST1, ZIC1, and ZIC4 are maintained in a non-ossifying state. As cells transition into the osteogenic front, anti-osteogenic regulators such as TWIST1, LMX1B, and ALX4 are progressively downregulated, whereas pro-osteogenic factors including RUNX2, DLX5, and SP7 are upregulated (26). Integrated transcriptomic and epigenomic analyses further suggested that this transition is controlled by enhancer-linked gene regulatory networks that spatially restrict osteogenic differentiation to appropriate anatomical domains.

This developmental logic is relevant to adult bone remodeling because several of the same transcriptional regulators, including TWIST1, RUNX2, SP7, and DLX5, continue to define osteogenic competence in adult mesenchymal and osteoblast-lineage populations (27). Rather than viewing osteogenesis as a simple linear differentiation sequence, the osteogenic front illustrates how bone formation can be gated by spatially restricted permissive and inhibitory signals. Similar principles may operate at adult remodeling surfaces, where canopy-associated and endosteal progenitors are maintained in a poised state until they receive appropriate spatial and temporal cues for osteoblast differentiation and matrix deposition.

Thus, developmental skeletal atlases support the broader concept that bone formation is governed by spatially restricted molecular permissiveness. The BRC canopy should therefore not be interpreted simply as a passive anatomical covering, but as a potential adult remodeling structure that recapitulates some organizational principles of developmental osteogenic fronts: local containment of progenitors, controlled exposure to differentiation cues, and spatial restriction of matrix-forming activity.

### 5.3 Cell–cell communication in spatial context

A key advantage of integrating spatial transcriptomics with scRNA-seq is the ability to move beyond dissociation-based inference of ligand–receptor interactions toward spatially anchored models of cell–cell communication. In the adult human bone atlas, Lin et al. (2025) used spatial modeling to show that TGFβ signaling colocalized with fibroblasts, endothelial cells, and osteoblastic-lineage cells within the bone formation niche (12). This finding suggests that TGFβ may serve as a shared communication axis linking stromal, vascular, and osteogenic components of the niche. In parallel, the mutually overlapping spatial distributions of NFκB and TNFα signaling indicate that inflammatory pathways are not merely systemic modifiers of bone loss, but may be locally organized within the bone microenvironment.

Computational frameworks such as CellChat and CellPhoneDB have become increasingly important for reconstructing these communication networks from single-cell and spatial transcriptomic data (30, 31). When combined with anatomical coordinates, these approaches can help distinguish ligand–receptor interactions that are merely co-expressed in dissociated datasets from those that are spatially plausible within intact tissue. This distinction is particularly important in bone, where osteogenic cells, osteoclasts, endothelial cells, adipocytes, immune cells, and matrix-embedded osteocytes occupy sharply different physical compartments.

Complementary spatial analyses further support the idea that the bone formation niche integrates signals across multiple spatial scales. Qiu et al. (32) applied 10X Visium to mouse femur and adjacent skeletal muscle and identified signaling pathways involved in intra- and inter-tissue communication, including COLLAGEN, THBS, VEGF, FN1, and TENASCIN pathways (32). Spatially supported ligand–receptor pairs included Col1a2–Sdc4, reflecting osteoblast–matrix interactions; Comp–Sdc4, linking monocyte/macrophage populations to osteoblasts; Tnxb–Sdc4, suggesting muscle–endothelial communication; and Vegfa–Vegfr1/Vegfr2, connecting muscle-derived angiogenic signals with endothelial and myeloid compartments. Although these findings require further validation, they illustrate how spatial transcriptomics can uncover communication axes that would be difficult to resolve from dissociated single-cell data alone.

Taken together, spatial communication analyses shift the interpretation of the bone formation niche from a collection of neighboring cell types to an interactive tissue ecosystem. In this view, bone formation depends on the local alignment of osteogenic progenitors, vascular support, matrix-derived signals, immune modulation, and osteoclast-coupled remodeling cues. Disruption of these spatial communication networks may therefore represent a central mechanism by which the osteoporotic niche loses its capacity to support efficient bone formation. Representative cellular components, osteolineage trajectories, and spatial signaling gradients across this niche are summarized in **Figure 2**.

### 5.4 Disease and skeletal-site context of current human spatial atlases

The current human spatial datasets require careful interpretation in relation to osteoporosis. The samples analyzed by Lin et al. and Bandyopadhyay et al. were obtained from femoral heads removed during arthroplasty in patients with osteoarthritis (10, 12). Osteoarthritis and osteoporosis are biologically distinct disorders. Osteoarthritis is a joint-centered disease associated with inflammation, altered mechanical loading, subchondral bone sclerosis, and spatially heterogeneous bone remodeling (46), whereas osteoporosis is a systemic skeletal disorder characterized by reduced bone mass, microarchitectural deterioration, and increased fragility (1, 3). Osteoarthritis-associated changes may therefore influence stromal-cell states, inflammatory signaling, vascular organization, matrix mineralization, and the relative abundance of osteogenic and adipogenic populations. Consequently, these datasets should not be interpreted as direct molecular maps of the osteoporotic bone formation niche.

Nevertheless, these atlases remain valuable as reference maps because they identify spatial relationships that can generate testable hypotheses, including the enrichment of osteogenic stromal populations near trabecular surfaces and the spatial segregation of osteogenic and adipogenic marrow domains (10, 12). In the present framework, these observations are integrated with direct histomorphometric evidence of canopy loss and reversal-phase arrest in human osteoporosis (6, 7). Thus, the proposed spatial de-zonation framework represents a synthesis of direct histological evidence from osteoporosis and indirect spatial molecular evidence from reference human atlases and experimental models, rather than a spatial-transcriptomic demonstration of osteoporosis itself.

Generalizability across skeletal sites also remains uncertain. The femoral head is exposed to a distinct mechanical environment and differs anatomically and functionally from vertebral bodies, the distal radius, proximal humerus, and intertrochanteric femur, which are clinically important sites of osteoporotic fracture. Skeletal stem and progenitor populations exhibit site-specific identities (14–16), while cortical-to-trabecular ratios, marrow composition, vascular organization, mechanical loading, and remodeling dynamics vary among skeletal regions. Future studies should therefore compare spatial niche organization across clinically relevant fragility-fracture sites and include non-osteoarthritic controls and well-phenotyped osteoporotic cohorts.

## 6 A proposed spatial de-zonation framework for osteoporosis

As discussed above, current human spatial atlases provide reference maps rather than direct evidence of osteoporosis-specific spatial de-zonation. The following framework should therefore be regarded as a testable model that integrates these reference maps with histological evidence from osteoporotic bone.

### 6.1 Canopy disruption and loss of niche containment

The BRC canopy model provides a direct conceptual link between spatial organization and impaired bone formation in osteoporosis. Histomorphometric studies have shown that canopy coverage over remodeling sites is reduced in postmenopausal osteoporotic women, and that reduced canopy coverage is associated with a prolonged reversal phase and diminished bone formation (6). In glucocorticoid-induced osteoporosis, both canopy cells and the underlying reversal cells appear to be affected, suggesting a compounded defect in the cellular infrastructure required for coupled remodeling (7). This dual impairment may help explain why glucocorticoid exposure produces a particularly severe suppression of bone formation.

From a spatial perspective, canopy disruption is not merely the loss of a surface-covering structure. It represents the breakdown of a specialized microenvironment that normally separates the remodeling surface from the surrounding marrow, concentrates osteoprogenitors near sites of prior resorption, and supports the transition from reversal to formation. When this structure is compromised, osteoclast-mediated resorption may no longer be efficiently followed by osteoblast recruitment, differentiation, and matrix deposition. Thus, canopy disruption can be interpreted as an early lesion of spatial niche failure in osteoporosis.

It is important, however, to distinguish histological evidence from the newer spatial transcriptomic atlases discussed above. Current spatial atlases of adult human bone, including those generated from osteoarthritis femoral head samples, do not directly represent osteoporotic bone (12). Similarly, existing spatial proteomic datasets provide valuable reference maps but do not yet define disease-specific spatial remodeling in osteoporosis (10). These atlases should therefore be viewed as baseline frameworks against which osteoporotic niche disruption can be measured. A key next step will be to apply spatial transcriptomics and spatial proteomics directly to osteoporotic bone samples, ideally from fragility fracture sites or well-characterized osteoporotic cohorts, to determine whether the spatial gradients observed in reference bone are eroded, displaced, or transcriptionally reprogrammed in disease.

### 6.2 Osteolineage depletion and erosion of osteogenic territory

Age-related depletion of osteoblast-lineage populations can be reinterpreted through the lens of spatial organization. Zhou et al. (2025) identified distinct osteoblast-lineage cell populations with compartment-specific features, including marrow/endosteal OLC1 and periosteal OLC2 populations (27). These populations are not simply different maturation states; they appear to serve different anatomical regions and may contribute to different aspects of skeletal maintenance. The preferential loss of OLC2-associated extracellular matrix programs in aged bone suggests that periosteal osteogenesis may be particularly vulnerable during skeletal aging, potentially contributing to cortical thinning and increased porosity.

At the molecular level, reduced expression of lineage-associated transcription factors, including Sp7, Mef2c, and Creb3l1 in OLC1, and Prrx2, Maf, and Shox2 in OLC2, suggests that aging may impair not only osteolineage cell abundance but also osteogenic competence (27). In this context, osteolineage depletion should not be viewed solely as numerical cell loss. It may also reflect a failure to maintain the transcriptional programs that define spatially specialized osteogenic compartments.

We propose the term spatial de-zonation to describe the erosion of spatial gradients that normally separate osteogenic, vascular, and adipogenic domains within the marrow microenvironment. In healthy bone, osteogenic programs are concentrated near the bone surface, vascular and perivascular signals occupy transitional regions, and adipogenic programs are more prominent in the deeper marrow. With aging and osteoporosis, this organization may become blurred. Loss of osteoblast-lineage cells from the bone surface would be expected to weaken the identity of the osteogenic territory, reducing the capacity of the surface niche to support matrix deposition and mineralization.

This process may be particularly important because bone formation depends not only on the presence of osteogenic cells, but also on their correct spatial positioning. If osteoblast-lineage cells are depleted from the bone surface or lose their compartment-specific transcriptional identity, the local niche may fail to sustain the molecular gradients required for efficient osteogenesis. In this model, osteoporosis involves not only reduced osteoblast number or function, but also erosion of the osteogenic territory itself.

**Figure 3:**
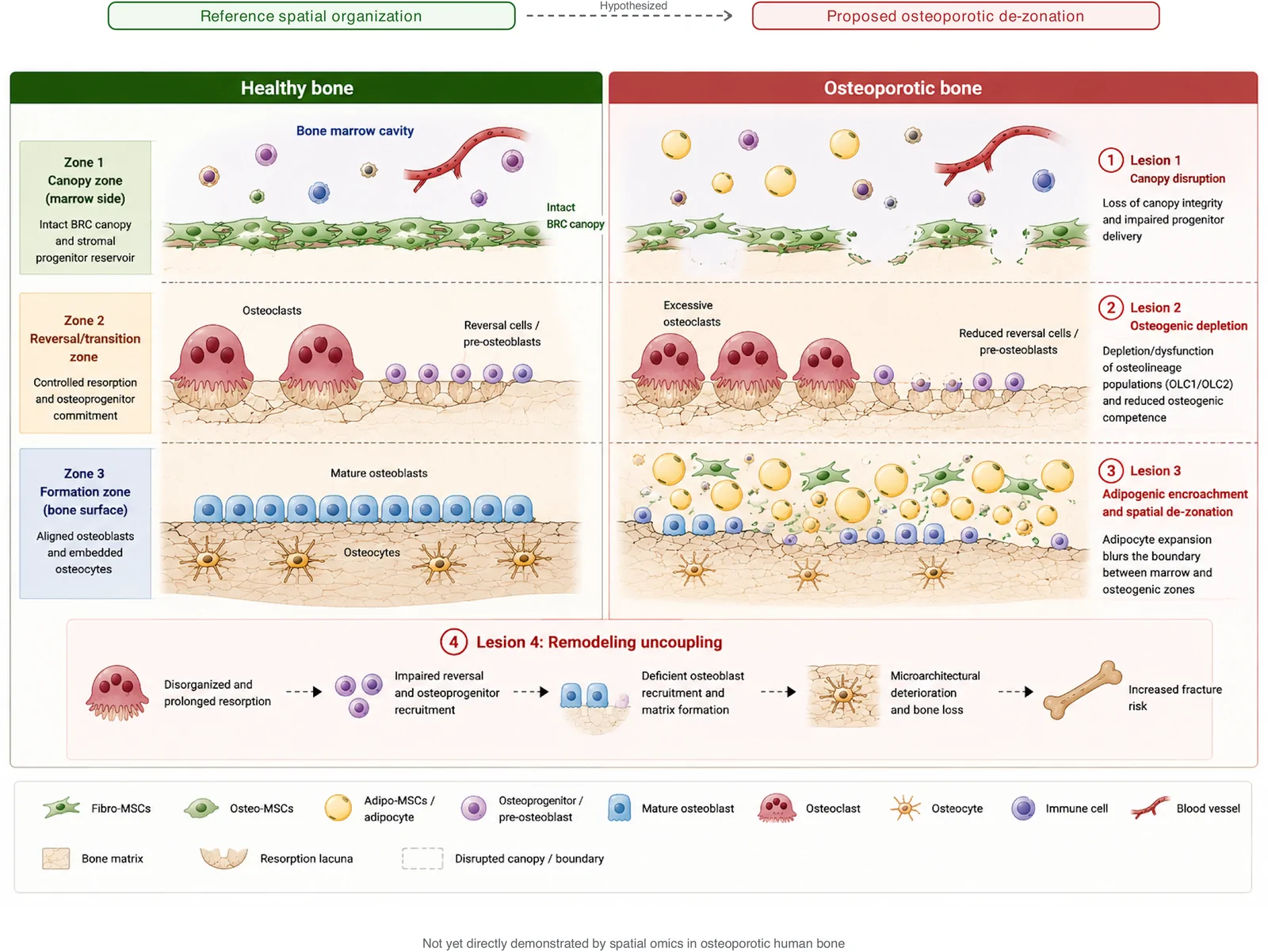
Proposed spatial de-zonation of the bone formation niche in osteoporosis. The healthy/reference organization is synthesized primarily from histological studies, spatial atlases of osteoarthritic femoral heads, and experimental models. The osteoporotic panel represents a hypothesis derived from these reference datasets together with direct histomorphometric evidence of canopy disruption and impaired reversal-to-formation transition in osteoporosis. Osteoporosis-specific spatial transcriptomic validation remains required.

This spatial de-zonation model is illustrated in **Figure 3**, which depicts the progressive erosion of the osteogenic surface niche, expansion of adipogenic marrow programs, and collapse of the spatial gradients required for efficient bone formation.

### 6.3 Adipogenic encroachment and remodeling of marrow space

In the spatial niche framework proposed above, adipogenic stromal cells, mature adipocytes, and lipid metabolic programs are normally enriched in the marrow-side adipogenic compartment adjacent to the canopy–stromal zone. With aging and estrogen deficiency, these adipogenic features may expand toward regions that normally support bone formation, creating a spatially disorganized microenvironment in which osteogenic signals are weakened or displaced.

This model is supported indirectly by several observations. Adipo-MSC signatures are enriched in aged bone marrow, and aging-related changes in MSC fate regulation shift the balance between osteogenic and adipogenic differentiation (10, 33, 34). Bone marrow adipose tissue is also increasingly recognized as an active regulator of skeletal homeostasis rather than a passive filler. Under estrogen-deficient conditions, BMAT expansion may suppress osteogenesis through altered adipokine secretion, fatty acid metabolism, paracrine signaling, and competition for progenitor fate allocation (30). These mechanisms provide a plausible biological basis for adipogenic encroachment into osteogenic territories.

The recently characterized Adipoq^+^Osx^+^ progenitor population further illustrates how adipogenic and osteogenic programs may intersect within the endosteal niche. These cells exhibit bipotent adipo-osteogenic potential, and estrogen deficiency accelerates the generation of marrow adipocytes from Osx^+^ progenitors (29). This finding suggests that the increase in marrow adiposity after menopause may not simply reflect expansion of a pre-existing adipocyte pool, but may also involve fate redirection of progenitors that are spatially positioned near bone-forming surfaces. Such fate shifts could directly weaken the osteogenic niche while simultaneously increasing local adipogenic influence.

Recent work on estrogen-related receptor alpha provides an additional mechanistic link between adipocyte-rich marrow and impaired osteogenesis. Huang et al. (2024) identified ESRRA as a mediator of the adipo-osteogenic switch in adipocyte-rich bone marrow (35). Adipocyte-specific ESRRA deletion preserved osteogenesis and type H vessel formation under estrogen deficiency and obesity. Mechanistically, ESRRA interfered with E2/ESR1 signaling, repressed Spp1, and increased leptin expression, thereby promoting a local adipocyte-derived program that suppresses bone formation (35). Pharmacological inhibition of ESRRA protected obese mice from bone loss and marrow adiposity, suggesting that adipocyte-derived niche-disrupting signals may be therapeutically targetable.

Together, these findings support a model in which osteoporosis involves progressive remodeling of marrow space. As osteogenic territories lose cellular and transcriptional identity, adipogenic signals may expand, progenitor fate may shift toward adipocytes, and the boundary between bone-forming and marrow-filling domains may become less distinct. This adipogenic encroachment represents a key component of spatial de-zonation in the osteoporotic niche.

### 6.4 Aberrant osteoclast positioning and defective coupling

Spatial dysregulation in osteoporosis may also involve osteoclasts and their coupling to osteoprogenitor recruitment. In physiological remodeling, osteoclasts do more than resorb bone; they initiate a spatially ordered sequence in which resorption is followed by reversal, progenitor recruitment, and osteoblast-mediated matrix deposition. This sequence requires not only the correct cell types, but also their positioning at the correct surface and at the correct time.

Recent spatial data from fracture healing models provide insight into how this coordination may deteriorate with aging. Noom et al. (2025, preprint) combined scRNA-seq with multi-epitope ligand cartography to map cellular organization during early fracture repair in young and aged mice (36). In young mice, activated osteoclasts were spatially concentrated within the cortical bone compartment at day 7 after fracture. A distinct Spp1-high macrophage subset, restricted to the cortex, was identified as a transitional precursor population giving rise to osteoclasts. Neutrophils preceded the accumulation of these Spp1-high macrophages, suggesting a temporally and spatially ordered neutrophil–macrophage–osteoclast sequence.

In aged mice, this spatial coordination was attenuated. Although individual cell states were partly preserved, the sequential recruitment and positioning of neutrophils, macrophages, and osteoclasts within the cortical niche were less clearly organized (36). This finding is important because it suggests that aging may impair remodeling not only by altering cell identity, but also by disrupting the spatial orchestration of otherwise recognizable cell states.

This concept aligns with the threshold model proposed by Delaissé et al. (2020), in which successful transition from resorption to formation requires the accumulation of sufficient osteoprogenitors on the eroded surface (21). If aging or osteoporosis disrupts the spatial signals that position osteoclasts, reversal cells, and osteoprogenitors, the remodeling sequence may fail to reach the cellular density and spatial organization required for bone formation. Aberrant osteoclast positioning may therefore contribute to uncoupled remodeling, not simply by increasing resorption, but by degrading the spatial template on which subsequent formation depends.

### 6.5 Sex-specific regulation of the bone formation niche

Osteoporosis is strongly sexually dimorphic, and the organization and failure of the bone formation niche are therefore unlikely to be identical in females and males (47, 48). In women, the abrupt decline in estrogen after menopause accelerates bone remodeling, enhances osteoclastogenic and inflammatory signaling, promotes marrow adiposity, and impairs osteoblast-lineage maintenance (30, 35, 47). These changes may affect several components of the proposed spatial niche simultaneously, including reversal-to-formation coupling, osteogenic progenitor retention, vascular support, and the boundary between osteogenic and adipogenic marrow domains. Whether estrogen deficiency directly disrupts canopy integrity remains unknown. However, the estrogen-deficiency-induced redirection of endosteal Adipoq^+^Osx^+^ progenitors toward adipocytes provides a potential cellular mechanism linking systemic sex-hormone loss to local spatial de-zonation (29).

Male skeletal aging follows a different hormonal and structural trajectory. Both androgen receptor signaling and estrogen generated through aromatization contribute to male bone homeostasis, whereas age-related declines in sex steroids are generally more gradual than the menopausal transition (47, 48). Sex differences in skeletal size, cortical geometry, and periosteal apposition are well established (48), while differences in marrow adiposity, immune responses, muscle-derived mechanical loading, and progenitor-cell abundance may further modify how niche dysfunction develops and influences fracture susceptibility (29, 30, 47). Consequently, the relative contributions of canopy failure, progenitor depletion, adipogenic encroachment, and remodeling uncoupling may differ between women and men and between cortical and trabecular compartments. However, direct spatial evidence for these proposed sex-dependent differences remains limited.

Current single-cell and spatial datasets are not sufficiently powered to resolve these differences. Available human atlases generally include small numbers of arthroplasty specimens and frequently lack stratification by sex, menopausal status, circulating sex hormones, osteoporosis diagnosis, and prior treatment (10, 12). Future spatial studies should therefore be prospectively designed to include both sexes, report these clinical variables, and test sex-by-age and sex-by-skeletal-site interactions. Until such data become available, the spatial de-zonation framework should not be assumed to apply identically to female and male osteoporosis.

### 6.6 Clinical implications of spatial niche failure

A spatial perspective provides a useful framework for understanding several clinical features of osteoporosis and its treatment. First, antiresorptive therapies can effectively suppress osteoclast activity and reduce fracture risk, but they do not necessarily restore bone formation. From a niche perspective, this is expected. If the canopy is disrupted, osteoprogenitors are depleted, or osteogenic and adipogenic domains are spatially disorganized, suppressing resorption alone may not be sufficient to rebuild the cellular architecture required for new bone formation.

Second, the parallel increase in marrow adiposity and decline in bone mass during aging and estrogen deficiency can be interpreted as spatial remodeling of the marrow environment. Rather than representing two independent processes, adipocyte expansion and osteolineage decline may reflect a shared disruption of progenitor fate and niche organization. Expansion of Adipo-MSCs, marrow adipocytes, or bipotent Adipoq^+^Osx^+^ progenitor-derived adipocytes may weaken the spatial dominance of the osteogenic surface niche and shift the marrow toward an adipogenic state (29).

Third, the plateauing of anabolic responses to agents such as teriparatide and romosozumab may reflect, at least in part, constraints imposed by niche capacity. Anabolic signaling can stimulate osteoblast activity, but sustained bone formation may still require an intact spatial infrastructure: available osteoprogenitors, vascular support, matrix cues, canopy-associated organization, and limited adipogenic interference. Once these supporting elements become limiting, stronger anabolic stimulation may not produce proportionally greater bone formation.

This interpretation does not diminish the clinical value of existing antiresorptive or anabolic therapies. Rather, it suggests that future osteoporosis treatment may need to consider not only which pathway is activated or inhibited, but also whether the spatial architecture of the bone formation niche can be preserved or restored. In this sense, osteoporosis can be viewed not only as a disease of cellular imbalance, but also as a disease of niche disorganization.

## 7 Discussion

### 7.1 Therapeutic implications: from cell-targeted therapy to niche restoration

The spatial reconceptualization of the bone formation niche has important therapeutic implications. Current anabolic therapies, including parathyroid hormone analogs and anti-sclerostin antibodies, primarily act by stimulating osteoblast activity, promoting osteoprogenitor differentiation, or enhancing bone-forming signals (4, 37). However, if the underlying niche infrastructure is compromised—through canopy disruption, osteoprogenitor depletion, loss of transcriptional competence, or adipogenic encroachment—then anabolic stimulation is applied to a degraded microenvironment with limited capacity to sustain bone formation.

**Figure 4:**
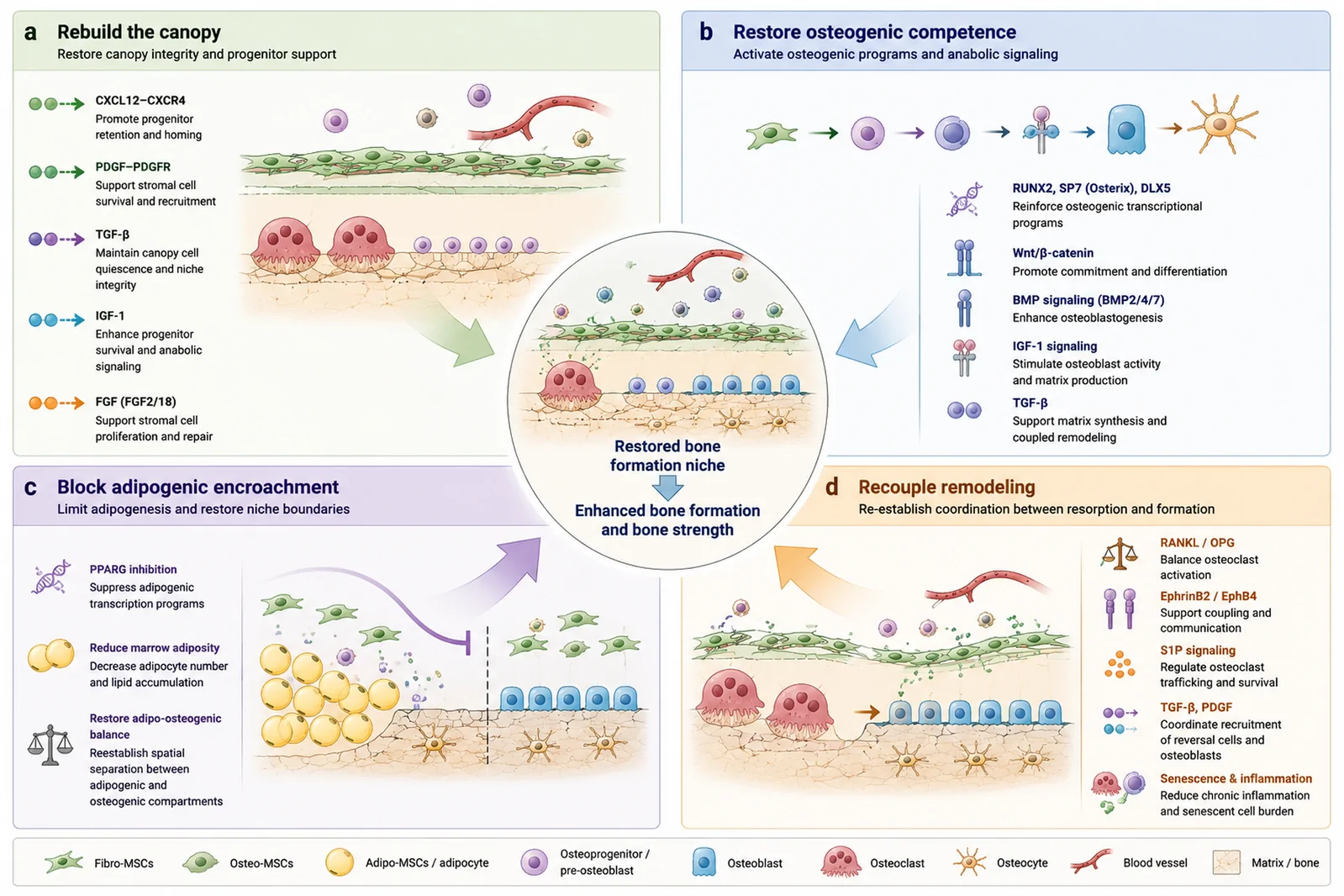
Niche-restorative strategies for osteoporosis. This schematic summarizes four therapeutic axes aimed at restoring the spatial integrity and functional competence of the bone formation niche. **(a)** Rebuilding the BRC canopy may enhance canopy integrity, progenitor retention, and stromal support through CXCL12–CXCR4, PDGF–PDGFR, TGF-β, IGF-1, and FGF signaling. **(b)** Restoring osteogenic competence may reactivate osteogenic transcriptional programs and anabolic pathways, including RUNX2, SP7/Osterix, DLX5, Wnt/β-catenin, BMP, IGF-1, and TGF-β signaling. **(c)** Blocking adipogenic encroachment may limit PPARG-driven adipogenesis, reduce marrow adiposity, and restore the boundary between adipogenic and osteogenic compartments. **(d)** Recoupling remodeling may re-establish coordination between osteoclast activity, reversal cell recruitment, and osteoblast-mediated bone formation through RANKL/OPG, ephrinB2/EphB4, S1P, TGF-β, PDGF, and senescence- or inflammation-related pathways.

A niche-oriented therapeutic strategy would therefore aim not only to activate osteoblast-lineage cells, but also to restore the spatial conditions that allow these cells to function. Such an approach may involve re-establishing canopy integrity, recruiting progenitors back to the bone surface, preserving osteogenic transcriptional programs, limiting adipogenic invasion of the marrow space, and restoring the spatial coupling between resorption and formation. **Figure 4** summarizes four interconnected therapeutic axes that correspond to the major spatial lesions discussed in this review: rebuilding the BRC canopy, reactivating osteogenic competence, blocking adipogenic encroachment, and restoring remodeling-coupled spatial organization.

The Wnt/β-catenin pathway, with sclerostin as its endogenous inhibitor, is currently the most clinically successful example of manipulating a spatially embedded bone-forming pathway. Romosozumab, a humanized monoclonal antibody against sclerostin, promotes bone formation while reducing bone resorption by releasing Wnt signaling from sclerostin-mediated inhibition (31, 38). However, its anabolic effect is time limited, and treatment duration is generally restricted because the bone-forming response wanes with continued therapy (38). From a spatial niche perspective, this plateau may reflect not only pathway-level feedback, but also the finite capacity of the available osteoprogenitor reservoir, canopy-associated microenvironment, and vascular–stromal support system to sustain continued osteogenesis.

This interpretation also highlights a spatial disconnect inherent to anti-sclerostin therapy. Sclerostin is produced predominantly by osteocytes embedded within the bone matrix, whereas the desired anabolic response occurs mainly at bone-forming surfaces where osteoprogenitors and osteoblast-lineage cells are located (31). Thus, effective Wnt activation requires signal propagation across spatially distinct compartments: from matrix-embedded osteocytes to surface-associated osteogenic cells. Ageing or osteoporosis-related disruption of osteocyte function, canopy integrity, or osteoprogenitor positioning may therefore influence the magnitude and durability of the therapeutic response.

Beyond sclerostin blockade, several molecular and cellular targets discussed in this review may support future niche-restorative strategies. osteostaticytes, through their CCL3/CCL4-mediated MSC chemotactic activity, represent a potential mechanism for enhancing progenitor recruitment to remodeling surfaces (28). The TWIST1–RUNX2 regulatory axis and related developmental programs may help define the transcriptional switch that permits progenitor-to-osteoblast transition (26). SOX4-mediated regulation of osteogenic competence further points to transcriptional control as a therapeutic entry point (39). The VDR–SOD2–ROS axis links oxidative stress, MSC senescence, and impaired osteogenic differentiation, suggesting that maintenance of stromal cell fitness may be essential for preserving niche function during aging (34). Similarly, the aging-vulnerable OLC2 lineage and its extracellular matrix programs may provide a compartment-specific target for strategies aimed at periosteal bone formation (27).

Adipocyte-derived signals represent another important therapeutic axis. The identification of ESRRA as a mediator of adipocyte-driven suppression of osteogenesis suggests that targeting adipocyte-rich marrow environments may help counteract adipogenic encroachment and preserve osteogenic niche identity (35). Likewise, pathways regulating osteoclast differentiation and positioning, including the Pla2g7–Alox12/12-HETE/Gpr31 signaling axis, may provide opportunities to restore remodeling-coupled spatial organization rather than merely suppressing resorption (40). Together, these emerging targets suggest that future osteoporosis therapies may need to move beyond activating or inhibiting individual cell types, toward restoring the spatial architecture that enables coordinated bone formation.

### 7.2 Key open questions and future research agenda

Several fundamental questions need to be addressed before the concept of spatial niche restoration can be translated into osteoporosis therapy. First, the molecular identity of the BRC canopy remains incompletely defined. Although histological and immunophenotypic studies have characterized canopy-associated cells, it is still unclear whether the canopy possesses a distinct transcriptomic signature that separates it from adjacent bone-lining cells, stromal progenitors, and reversal-phase cells. Resolving this question will require higher-resolution spatial platforms, such as MERFISH, Xenium, or other imaging-based approaches, ideally combined with bone-optimized tissue processing and targeted gene panels.

Second, it remains uncertain whether the bone formation niche can be activated independently of prior bone resorption. Physiological bone remodeling is tightly coupled: osteoclast-mediated resorption is followed by reversal, progenitor recruitment, and osteoblast-mediated formation. If osteostaticytes, reversal cells, or osteoclast-derived signals are required to recruit and activate osteoprogenitors, then purely anabolic strategies may be limited unless they also restore the coupling machinery that normally precedes bone formation. Understanding whether bone formation can be uncoupled from resorption without compromising spatial organization will be crucial for designing next-generation anabolic therapies.

Third, osteoporosis-specific spatial alterations should be directly compared across clinically relevant skeletal sites, sexes, and age groups using non-osteoarthritic controls and well-phenotyped osteoporotic cohorts. These studies will be essential for distinguishing universal niche features from disease-, site-, and sex-specific alterations.

Fourth, cross-species translation requires systematic evaluation. Mouse models such as ovariectomy, aging, glucocorticoid exposure, and immobilization remain indispensable for mechanistic and therapeutic studies, but it is unclear how faithfully they recapitulate the spatial gradients observed in human bone. Comparative spatial transcriptomics and spatial proteomics across species will be needed to identify conserved niche programs and to distinguish them from species-specific or site-specific features.

Fifth, future studies should determine whether spatial niche pathology can be inferred non-invasively. Direct spatial transcriptomic profiling of human bone will remain constrained by tissue accessibility and the need for invasive sampling. An important translational goal is therefore to develop imaging, circulating, and computational biomarkers that reflect spatial niche integrity. High-resolution peripheral quantitative computed tomography, magnetic resonance imaging of marrow adiposity, serum markers of bone turnover, and spatially informed molecular signatures could eventually be integrated to approximate niche status in living patients. Such approaches may provide a basis for spatial precision medicine in osteoporosis, in which treatment selection is guided not only by bone mineral density and conventional fracture-risk assessment but also by the inferred state of the bone formation niche.

Finally, the field will need experimental systems capable of testing niche-restorative interventions directly. These may include ex vivo human bone explants, bone-on-chip systems, organoid-like marrow models, and spatially resolved perturbation approaches that combine lineage tracing, genetic manipulation, and spatial omics. Such platforms will be necessary to determine whether restoring canopy integrity, re-establishing osteogenic gradients, limiting adipogenic encroachment, or improving osteoclast–osteoblast coupling can meaningfully enhance bone formation in aged or osteoporotic bone.

In summary, the therapeutic implication of the spatial niche model is not that existing osteoporosis therapies are inadequate, but that their efficacy may depend on the architectural state of the tissue in which they act. Future strategies may therefore combine pathway activation with restoration of the spatial microenvironment required for durable bone formation.

## 8 Concluding remarks

The convergence of single-cell and spatial transcriptomic technologies is reshaping how bone formation is understood. Bone biology has traditionally focused on identifying relevant cell types and defining their molecular states. Recent spatial atlases extend this view by showing that bone formation also depends on where these cells are positioned, how they interact, and whether their organization is preserved within a specialized tissue microenvironment.

This spatial perspective provides a molecular extension of the BRC canopy concept. The close association of RUNX2^+^ osteoblastic-lineage cells, stromal fibroblast-like populations, matrix-associated programs, and locally organized signaling pathways at bone surfaces supports a model in which bone formation is governed by spatially restricted niche activity rather than by linear differentiation alone.

For osteoporosis, this framework reframes bone loss as more than a simple imbalance between osteoblasts and osteoclasts. Canopy disruption, osteolineage depletion, adipogenic encroachment, and defective remodeling coupling together suggest that osteoporosis may also be understood as a disease of niche disorganization. The critical next step is to determine whether these spatial lesions can be directly visualized and molecularly characterized in osteoporotic human bone using bone-compatible processing methods and high-resolution spatial platforms.

Ultimately, the spatial niche model does not replace existing cellular and molecular paradigms, but integrates them into a tissue-level framework. Future therapies may need not only to stimulate osteoblasts or suppress osteoclasts, but also to preserve or restore the architectural integrity of the osteogenic niche itself.
