# Supplementary material for: Bone formation niche dysfunction in osteoporosis: insights from single-cell and spatial transcriptomic studies
Source: Front Endocrinol (Lausanne). 2026 Aug 26;17:1909423. doi: 10.3389/fendo.2026.1909423 (PMC13562837; doi:10.3389/fendo.2026.1909423)
Supplement: Supplementary file 1 [file SupplementaryFile1.docx]

**Supplementary File**

**Reconstructed AI Prompts and Responses Used in Manuscript Preparation**

| **Manuscript title** | Bone Formation Niche Dysfunction in Osteoporosis: Insights from Single-Cell and Spatial Transcriptomic Studies |
| --- | --- |
| **Manuscript type** | Review |
| **Authors** | Tingting Tan; Xiaoning Guo; Zhengxiao Ouyang |
| **AI system** | OpenAI conversational generative-AI system. The exact interface, model/version, and dates of use were not retained. |

# Important transparency statement

# The original AI chat history was not retained. Accordingly, the material below is a retrospective reconstruction based on the current revised manuscript and the authors’ recollection of the types of AI assistance used. It is not a verbatim transcript and should not be interpreted as a complete log of every interaction. The wording of both prompts and responses has been reconstructed to represent their substance as faithfully as possible. No source citation, numerical value, or scientific claim should be attributed to the AI system solely on the basis of this file.

# Scope of AI assistance and author oversight

- AI assistance was used for drafting or restructuring selected passages, preparation of tables and figure-caption concepts, identification of statements requiring caution, and English-language editing.
- The authors selected the literature, checked the cited publications against source material, evaluated scientific accuracy, revised all AI-assisted text, and determined the final interpretations and conclusions.
- The AI system did not determine authorship, independently perform experiments or analyses, assume responsibility for the work, or replace author judgment. The authors accept full responsibility for the manuscript.

# Reconstructed prompt–response record

*The entries below are grouped by substantive task rather than chronology. “Reconstructed AI response” records the substance of the assistance reflected in the revised manuscript; it is not presented as verbatim model output.*

## Interaction 1. Summarizing technical challenges in bone spatial transcriptomics

**Reconstructed prompt**

Summarize why single-cell and spatial transcriptomic profiling of mineralized bone is difficult. Compare EDTA decalcification with rapid acid-based approaches, explain spot/bin resolution and RNA-quality limitations, and keep the wording technically cautious.

**Reconstructed AI response**

Mineral removal is required for cell isolation or tissue sectioning, but prolonged decalcification can degrade RNA and distort transcript recovery. EDTA is gentle to morphology but slow, whereas rapid acid-based decalcification can shorten processing time and improve RNA preservation when carefully optimized. Sequencing-based spatial platforms trade spatial resolution against transcript detection, and each spot or bin may contain mixed cell types. Imaging-based methods provide higher spatial precision but depend on a predefined target panel. Bone-specific workflows must also prevent section detachment and account for tissue autofluorescence and matrix-related artifacts. Comparisons across platforms should therefore consider specimen type, decalcification chemistry, RNA quality, resolution, sensitivity, and compatibility with downstream validation.

**Author review and use**

The authors critically reviewed this material, checked relevant statements against the cited literature, revised the wording and interpretation, and retained only content they judged appropriate for the final manuscript.

## Interaction 2. Preparing the evidence-summary table

**Reconstructed prompt**

Create a table template for representative studies informing the bone formation niche framework. Include columns for study, approach/platform, sample or model, key contribution, disease/site context and interpretive limitation, and reference. Emphasize limitations such as osteoarthritis rather than osteoporosis, mouse-to-human translation, developmental tissue, spot-level cell mixing, and two-dimensional histology.

**Reconstructed AI response**

Recommended columns are: Study; Approach/Platform; Sample/Model; Key contribution to the spatial niche framework; Disease/site context and interpretive limitation; and Reference. Each row should state both what the study contributes and what it cannot establish. Human femoral-head atlases should be identified as osteoarthritis/arthroplasty reference maps rather than osteoporosis cohorts. Mouse lineage-tracing studies can strengthen causal interpretation but remain subject to species and skeletal-site differences. Developmental atlases reveal organizing principles but cannot be directly equated with adult remodeling. Visium spots may mix several cell types, while histomorphometric canopy studies are anatomically relevant but largely two-dimensional and correlative.

**Author review and use**

The authors critically reviewed this material, checked relevant statements against the cited literature, revised the wording and interpretation, and retained only content they judged appropriate for the final manuscript.

## Interaction 3. Preparing the spatial-cell table

**Reconstructed prompt**

Create a concise table template summarizing spatially organized cellular constituents of the bone formation niche. Use columns for cell type, key markers, niche location, putative function, and references. Include Fibro-MSC, Osteo-MSC, Adipo-MSC, Adipoq-positive/Osx-positive progenitors, BME/canopy cells, mature osteoblasts, osteostaticytes, osteocytes, and osteoclasts.

**Reconstructed AI response**

The table should map each population to the proposed functional zones while avoiding claims of rigid boundaries. Fibro-MSCs can be assigned to the canopy/marrow-side stromal region as primitive stromal and extracellular-matrix-supporting cells. Osteo-MSCs occupy canopy-proximal or reversal-associated osteogenic regions. Adipo-MSCs lie in a neighboring adipogenic marrow compartment. Adipoq-positive/Osx-positive cells represent an endosteal bipotent progenitor population. BME/canopy cells may support segregation and progenitor delivery. Osteoclasts and osteostaticytes belong primarily to the resorption/reversal domain, whereas mature osteoblasts and osteocytes define the formation and mineralized-matrix domains. Marker lists and functions should be checked against the cited primary studies.

**Author review and use**

The authors critically reviewed this material, checked relevant statements against the cited literature, revised the wording and interpretation, and retained only content they judged appropriate for the final manuscript.

## Interaction 4. Developing figure concepts and captions

**Reconstructed prompt**

Propose four schematic figures for the review: (1) healthy versus disrupted BRC canopy, (2) the cellular and spatial architecture of the bone formation niche, (3) spatial de-zonation in osteoporosis, and (4) niche-restorative therapeutic strategies. Provide caption-level descriptions and clearly label the osteoporosis de-zonation panel as a hypothesis.

**Reconstructed AI response**

Figure 1 may contrast an intact canopy-covered remodeling compartment with canopy disruption, inadequate progenitor recruitment, and reversal-phase arrest. Figure 2 may depict the three functional zones, their major cellular constituents, osteolineage progression, and representative signaling gradients. Figure 3 should contrast the reference organization with a proposed osteoporotic state in which stromal, osteogenic, vascular, and adipogenic gradients become less distinct; its caption must state that osteoporosis-specific spatial transcriptomic validation is lacking. Figure 4 may organize therapeutic concepts into rebuilding the canopy/stromal infrastructure, restoring osteogenic competence, limiting adipogenic encroachment, and recoupling resorption with formation. All pathways shown should be treated as candidates unless directly validated in the relevant niche context.

**Author review and use**

The authors critically reviewed this material, checked relevant statements against the cited literature, revised the wording and interpretation, and retained only content they judged appropriate for the final manuscript.

## Interaction 5. Drafting therapeutic implications and future directions

**Reconstructed prompt**

Organize the therapeutic implications of the niche model without implying that proposed targets are clinically validated. Include niche reconstruction, osteogenic competence, adipogenic encroachment, remodeling recoupling, spatially resolved human cohorts, causal perturbation, and multimodal validation.

**Reconstructed AI response**

The niche perspective suggests that future therapies might aim to restore architecture as well as alter individual cell activity. Candidate axes include stabilizing canopy/stromal support, maintaining osteoprogenitor retention, reactivating osteogenic transcriptional programs, preventing expansion of adipogenic marrow domains, and restoring coupling signals between osteoclast-lineage and osteoblast-lineage cells. These concepts remain largely preclinical or hypothesis-generating. Priority studies include osteoporosis-specific human spatial atlases with sex, age, skeletal site, treatment, and fracture-status annotation; lineage-specific perturbation of canopy-associated populations; spatially resolved ligand–receptor validation; and integration of transcriptomic maps with histomorphometry, imaging, Raman composition, and mechanical measurements.

**Author review and use**

The authors critically reviewed this material, checked relevant statements against the cited literature, revised the wording and interpretation, and retained only content they judged appropriate for the final manuscript.

## Interaction 6. Revising the abstract for calibrated claims

**Reconstructed prompt**

Edit the abstract of this review so that it highlights the three-zone niche model and spatial de-zonation concept while explicitly acknowledging that available human spatial atlases are mainly from osteoarthritic femoral heads and do not directly establish osteoporosis-specific spatial disorganization.

**Reconstructed AI response**

The abstract should frame the review as a synthesis of emerging evidence rather than a report of new experimental data. It may state that recent single-cell and spatial methods allow molecular mapping of bone-forming microenvironments, that the literature can be integrated into a three-zone model, and that spatial de-zonation is proposed as a hypothesis-generating framework. A key limitation sentence should specify that existing human spatial atlases predominantly derive from osteoarthritic femoral heads and therefore serve as reference maps rather than direct evidence of osteoporosis-specific niche disruption. The conclusion may emphasize testable questions and niche-restorative strategies without claiming clinical validation.

**Author review and use**

The authors critically reviewed this material, checked relevant statements against the cited literature, revised the wording and interpretation, and retained only content they judged appropriate for the final manuscript.

## Interaction 7. Final language and consistency editing

**Reconstructed prompt**

Edit the manuscript for scientific English, consistency, and cautious interpretation. Preserve technical terms and citations. Standardize single-cell RNA sequencing/scRNA-seq, spatial transcriptomics, BRC, BME, MSC, osteoblast-lineage cell, osteoporosis/OP, figure and table citations, hyphenation, and capitalization. Flag sentences that overgeneralize from mouse, developmental, osteoarthritis, or preprint evidence.

**Reconstructed AI response**

The editing pass should reduce repetition, shorten long sentences, define abbreviations at first use, and use consistent terms throughout. Statements derived from mouse, developmental, osteoarthritis, fracture-repair, or preprint studies should identify their context and avoid direct extrapolation to human osteoporosis. Words such as “demonstrates,” “establishes,” or “proves” should be reserved for appropriately supported findings; “suggests,” “supports,” “is consistent with,” or “provides a hypothesis” are preferable for inferential synthesis. Citations, numerical values, sample characteristics, marker identities, and platform details must be verified by the authors against the original sources.

**Author review and use**

The authors critically reviewed this material, checked relevant statements against the cited literature, revised the wording and interpretation, and retained only content they judged appropriate for the final manuscript.

# Author declaration

Because the original interaction logs were not retained, this file is provided as a transparent retrospective reconstruction in response to the editor’s request. The authors do not represent the reconstructed wording as a verbatim or exhaustive record. The authors take full responsibility for the selection and verification of references, scientific accuracy, originality, interpretation, and final content of the manuscript. The AI system is not an author and bears no responsibility for the work.
